# The Global Expansion of LTBI Screening and Treatment Programs: Exploring Gaps in the Supporting Economic Evidence

**DOI:** 10.3390/pathogens12030500

**Published:** 2023-03-22

**Authors:** Nokwanda Thandeka Kota, Suvesh Shrestha, Abdulhameed Kashkary, Pushpita Samina, Alice Zwerling

**Affiliations:** 1 Baylor College of Medicine Children’s Foundation eSwatini, Mbabane H100, Eswatini; 2 School of Epidemiology and Public Health, University of Ottawa, Ottawa, ON K1G 5Z3, Canada; 3 Public Health Authority, Riyadh 13351, Saudi Arabia; 4 Center for Health Economics and Policy Analysis, McMaster University, Hamilton, ON L8S 4L8, Canada

**Keywords:** LTBI, cost effectiveness, health economics, TB preventive treatment

## Abstract

The global burden of latent TB infection (LTBI) and the progression of LTBI to active TB disease are important drivers of ongoing TB incidence. Addressing LTBI through screening and TB preventive treatment (TPT) is critical in order to end the TB epidemic by 2035. Given the limited resources available to health ministries around the world in the fight against TB, we must consider economic evidence for LTBI screening and treatment strategies to ensure that limited resources are used to achieve the biggest health impact. In this narrative review, we explore key economic evidence around LTBI screening and TPT strategies in different populations to summarize our current understanding and highlight gaps in existing knowledge. When considering economic evidence supporting LTBI screening or evaluating different testing approaches, a disproportionate number of economic studies have been conducted in high-income countries (HICs), despite the vast majority of TB burden being borne in low- and middle-income countries (LMICs). Recent years have seen a temporal shift, with increasing data from low- and middle-income countries (LMICs), particularly with regard to targeting high-risk groups for TB prevention. While LTBI screening and prevention programs can come with extensive costs, targeting LTBI screening among high-risk populations, such as people living with HIV (PLHIV), children, household contacts (HHC) and immigrants from high-TB-burden countries, has been shown to consistently improve the cost effectiveness of screening programs. Further, the cost effectiveness of different LTBI screening algorithms and diagnostic approaches varies widely across settings, leading to different national TB screening policies. Novel shortened regimens for TPT have also consistently been shown to be cost effective across a range of settings. These economic evaluations highlight key implementation considerations such as the critical nature of ensuring high rates of adherence and completion, despite the costs associated with adherence programs not being routinely assessed and included. Digital and other adherence support approaches are now being assessed for their utility and cost effectiveness in conjunction with novel shortened TPT regimens, but more economic evidence is needed to understand the potential cost savings, particularly in settings where directly observed preventive therapy (DOPT) is routinely conducted. Despite the growth of the economic evidence base for LTBI screening and TPT recently, there are still significant gaps in the economic evidence around the scale-up and implementation of expanded LTBI screening and treatment programs, particularly among traditionally hard-to-reach populations.

## 1. Introduction

Latent TB infection (LTBI) with *Mycobacterium tuberculosis* (M.TB) affects approximately 25% of the globe. This large reservoir of potential active TB cases, coupled with scarce resources to support TB interventions, leads to an urgent need to find cost-effective and affordable LTBI screening and treatment approaches [1,2]. The resources required to scale up LTBI screening and TB preventive therapy (TPT) programs are sizeable, and many national TB programs, already struggling to provide active TB screening and treatment, do not have the funds or human resources required to scale up LTBI screening and TPT. The Stop TB Partnership estimates that over USD 209 billion is needed from 2023 to 2030 to end TB by 2030 [3]. The global scale-up of LTBI interventions has thus been slow and fraught with economic challenges. 

The fundamental utility of cost-effectiveness analyses lies in the guidance they provide to decision makers on how to effectively invest a limited pool of healthcare resources by allowing for the estimation and comparison of the cost per unit of health outcomes across different interventions. The importance of this exercise cannot be understated in TB programmatic planning, particularly regarding LTBI detection and treatment where resources are already highly constrained [4].

In this narrative review, we discuss key economic papers to explore our current understanding and gaps in our knowledge around the economic evidence to support the scale-up of TB screening and prevention programs. In practice, LTBI screening must be coupled with TPT in order to achieve health and economic gains; however, many of the economic evaluations around novel TPT regimens do not consider screening costs or approaches. Therefore, for the purposes of this review, we discuss economic evidence around LTBI screening and TPT in separate sections, noting overlap where appropriate.

**Figure 1:**
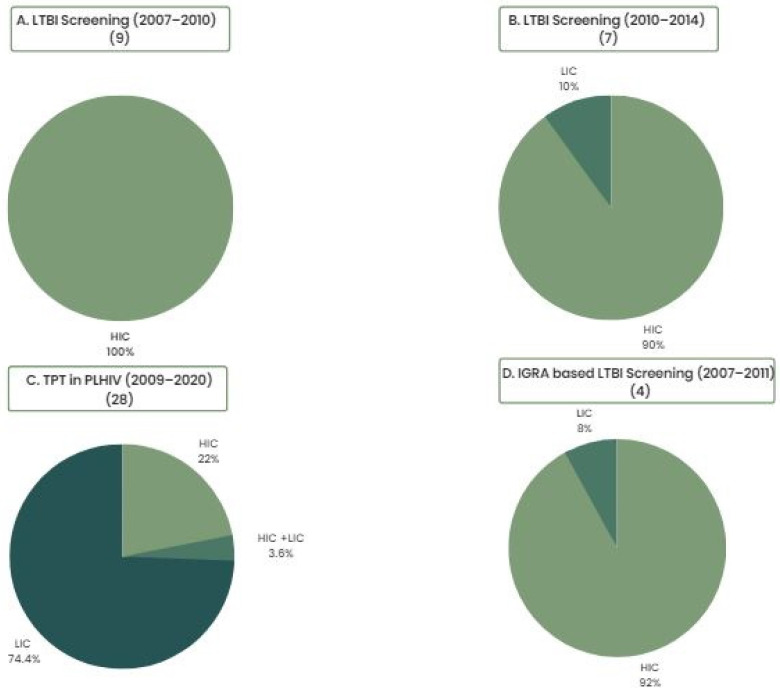
Systematic reviews of LTBI Screening and TPT cost-effectiveness analyses from the last 20 years (2007–2020): Depiction of the global temporal shift in prioritization and generation of economic evidence. HIC: high-income countries. LIC: low-income countries. (**A**) Nienhaus et al., 2011: Proportion of HIC- vs. LIC-conducted studies in a systematic review of cost-effectiveness analyses of different TB screening strategies from 2007 to 2010 [9]. (**B**) Auguste et al., 2016: Proportion of HIC- vs. LIC-conducted studies in a systematic review and economic evaluation of LTBI diagnosis in immunosuppression, children, and migrants from high-TB-incidence countries from 2010 to 2014 [7]. (**C**) Uppal et al., 2021: Proportion of HIC- vs. LIC-conducted studies in a systematic review of economic evidence for TPT in PLHIV from 2009 to 2020 [11]. (**D**) Oxlade et al., 2013: Proportion of HIC- vs. LIC-conducted studies in a systematic review of the methodological implications of IGRAs for LTBI diagnosis [4].

The overwhelming majority of economic evidence around LTBI screening and TPT has its origins in high-income, low-TB-burden countries. Evidence has, however, begun to be generated in low- and middle-income countries more recently, reflecting a global shift towards prioritization of LTBI screening and treatment (Figure 1) [4,5,6,7]. This is significant because economic evaluations are highly setting specific, and comparisons or generalizations should ideally be made with caution owing to differences in populations and health systems across different countries and settings, as well as variable modeling approaches and/or assumptions made across different studies. For example, the rate of progression to active TB has been shown to have an important impact on the cost effectiveness of LTBI interventions, meaning that the cost effectiveness of screening and TPT varies widely across different subpopulations with different risks of active TB progression. While implementation considerations may be more critical in ensuring the cost effectiveness of screening and TPT in the general population, economic evaluations have been highly consistent in demonstrating that screening and prevention programs for TB are highly cost effective when targeted towards contacts with recent TB exposure and PLHIV [4,8,9,10].

## 2. LTBI Screening

Diagnostic tests for LTBI are indirect, relying on the detection of an immune response to the M.TB antigen through either the tuberculin skin test (TST) or interferon-gamma release assays (IGRAs). While IGRAs possess higher specificity, particularly in BCG-vaccinated populations, compared with TST (95% specificity with TB-Spot, 97% with Quantiferon-TB Gold In-Tube test), they are a more costly diagnostic and require laboratory facilities and highly specialized training, unlike the low-cost in vivo TST approach which has been widely used globally for decades [12,13,14]. An economic systematic review published in 2011 found that costs of TST and IGRA varied widely across studies and countries, with TST costing anywhere from USD 7.4 in Japan to USD 145.99 in Germany, while IGRA was consistently more costly, ranging from USD 19 to USD 171.78 in the same settings [9].

TST has been the primary LTBI screening test used for over one hundred years due to its relatively low cost and logistical simplicity. The TST continues to remain a cost-effective LTBI screening modality in many settings and is used more extensively in low- and middle-income countries with high TB disease burden. Conversely, the use of IGRAs for LTBI screening is almost exclusively restricted to high- and upper-middle-income countries, with the more expensive upfront IGRA unit costs being offset by savings from costly targeted TB investigation and provision of TPT, which results in averted active TB cases and downstream savings [9,13,15].

In 2016, the U.S. Centers for Disease Control (CDC), through the U.S. Preventive Services Task Force, recommended that IGRAs replace TST in screening for LTBI in those with an increased risk for progression to active TB disease [14]. This decision was based partly on cost-effectiveness analyses, including one study from Linas et al. that demonstrated that IGRA is a cost-effective approach compared to TST alone in the U.S., particularly among close TB contacts and people living with HIV (PLHIV) [16].

**Figure 2:**
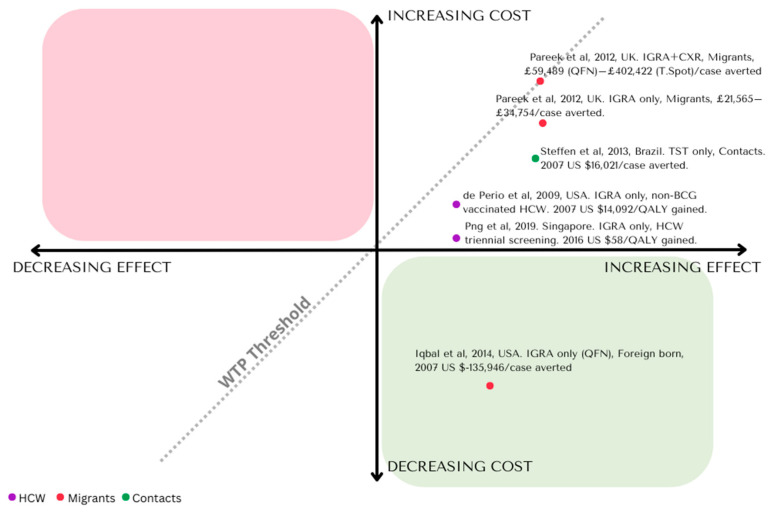
Incremental cost-effectiveness plane: illustration of modality specific ICERs for LTBI screening. Status quo modality including no screening, IGRA only, TST only, TST + IGRA, annual/biennial screening, etc. [9,15,19,20,21,22,23,24].

The National Institute for Health and Care Excellence (NICE) in the UK in 2016 recommended a sequential testing approach using IGRA to confirm a positive TST [17]. This sequential approach to LTBI testing was adopted by several European countries as well as Canada and was supported by several cost-effectiveness analyses including a UK study showing that a sequential testing strategy (TST followed by confirmatory IGRA) is more cost effective than either TST or IGRA alone. This conclusion is sensitive to changes in LTBI prevalence, the diagnostic accuracy of IGRAs and IGRA test costs, leading to wide disparities in the range of ICERs estimated from studies comparing LTBI screening approaches with IGRAs, visible on the cost-effectiveness plane in Figure 2 [7,18]. 

### 2.1. Screening among Migrants

Given the dominance of economic evidence coming from high-income countries, migrants are a key group often targeted for LTBI screening, with the bulk of economic assessments of LTBI screening in migrants coming from high-income countries (HICs) with low TB burden [19,25,26]. This trend has ancient origins because, as long ago as 10,000 years, TB is thought to have been transported to the Americas from Asia via migrants, while the pioneers are thought to have brought TB to New Zealand and Australia [27,28]. The LTBI screening strategies in migrant populations have included no screening, screening at the entry point, surveillance, screening contacts of TB patients and/or school screening in newly arrived immigrant children. An economic scoping review performed by Zammarchi et al. [26] in 2015 showed that, across the selected studies, targeted LTBI screening using IGRAs only in younger immigrants from endemic countries is generally a cost-effective approach. Of the reviewed studies, all were from HICs, four were conducted in the U.S., three in Canada and three in the UK. They compared screening with CXR, TST and TST followed by IGRA to each other and/or no screening, screening for active TB and contact investigation. The study results ranged from cost savings for IGRA strategies compared to TST only in immigrants in a U.S. study to a British study where IGRA-only screening costs an incremental GBP 21,565 to GBP 34,754 per averted TB case compared to a CXR and IGRA screening approach [26]. This, again, highlights the importance of setting, as IGRAs are a more resource-intensive test requiring laboratory infrastructure, laboratory capacity and specialized training. In settings where laboratory infrastructure is well developed and supported, implementing IGRAs may be cost effective or, as some studies have demonstrated, may even provide cost savings as they avert the costly downstream treatment costs associated with active TB disease. In other settings, implementing IGRAs may require building new laboratory infrastructure, buying and maintaining expensive laboratory equipment and more in-depth training of skilled laboratory personnel, leading to less favorable cost-effectiveness estimates.

Another key understanding has been the importance of targeting LTBI screening towards groups with recent exposure and/or higher risk of developing primary active disease to improve cost effectiveness. A study in Germany found LTBI screening is cost effective only among those from high-TB-incidence countries (ICER = EUR 51,000 per QALY gained) compared to screening at lower TB incidence thresholds [29].

### 2.2. Screening among Healthcare Workers

The vast majority of cost-effectiveness studies assessing LTBI screening and TPT among healthcare workers (HCWs) have also been performed in high-income, low-TB-incidence settings. That trend is starting to change. A 2019 study conducted on Brazilian primary healthcare workers found that TST is the most cost-effective LTBI screening strategy compared with IGRA or a sequential TST and IGRA testing approach [20]. While IGRAs have been shown to be cost effective in various high-income, low-TB-burden settings, it is the TST screening strategies that have been consistently more cost effective across most LMIC settings.

In addition to different diagnostic tests or algorithms, different frequencies of testing and key thresholds of risk for testing HCWs have also been areas of interest and research. In the intermediate-TB-burden setting of Singapore, Png et al. showed that targeted LTBI screening of high-risk HCWs every three years is the most cost-effective hospital screening strategy (ICER US $58 per QALY gained) compared to the status quo using CXR [21]. Another study in the European Union concluded that LTBI screening in HCWs is only cost effective under extremely high levels of increased risk for transmission [5]. The study found LTBI screening to be cost effective in Portugal (but not in the Netherlands) when assuming a relative force of infection (FOI) of 10 or 30 times higher than that of the low-risk population [5]. These studies nicely highlight the importance of context and comparators in economic evaluation. In settings where a costly CXR is the standard of care, introducing more expensive and more sensitive tests should lead to improved cost effectiveness. Likewise, where large groups of low-risk individuals are tested in non-differentiated screening campaigns, a more targeted approach can be cost effective and perhaps even provide cost savings.

In a low-TB-incidence setting, de Perio et al. showed that IGRA-only LTBI screening is a cost-effective approach in non-BCG-vaccinated U.S. healthcare workers (US $14,092 per QALY gained) compared to a TST-only approach, which is dominated by IGRA [9], but Mullie et al. concluded that annual LTBI screening in North American HCWs using IGRAs or TST is not cost effective due to the very small risk of annual TB infection [30]. 

### 2.3. Screening in High-Risk Populations

A recent systematic review of cost–utility analyses of LTBI screening tests specifically focused on high-risk populations, concluding that the economic evidence is robust enough to draw conclusions only for high-income and low-TB-burden countries, with limited evidence from high-TB-burden countries [10]. This systematic review also concluded that cost effectiveness varies depending on the LTBI prevalence or population tested; among low-risk populations, as well as children without any other LTBI or TB risk factors Screening with TST or no LTBI testing at all is often the most cost effective strategy compared to screening with IGRAs [7,10], reinforcing that LTBI screening should be carefully considered in low-TB-burden settings and only implemented for targeted high-risk groups who have increased risk of exposure and/or increased risk of progression to TB.

### 2.4. Screening in TB Contacts

Cost effectiveness studies from LMICs show that TST continues to be a cost-effective approach in most populations, including contacts of individuals with a TB diagnosis. A Brazilian study found a TST-only screening approach in TB contacts is the most cost-effective approach (US $16,021 per averted TB case) compared to a sequential TST/IGRA strategy (US $18,259 per averted TB case) or an IGRA-only approach (US $22,211 per averted TB case) [15]. Conversely, Kowada et al. found that an IGRA-only strategy is cost effective in close TB contacts in the intermediate-TB-incidence context of Japan (ICER = US $23,043 per QALY gained) compared to sequential TST and IGRA [31]. 

While heterogeneity exists in economic evidence around LTBI screening, studies are consistent in suggesting that IGRAs are likely cost effective in high-income settings, particularly when targeting groups with a high risk of progression to active TB. However, as TB prevalence or risk of active disease decreases in HICs, a TST-only or no-testing strategy may be preferred. Conversely, in LMICs with moderate- or high-TB-burden settings, TST is often more cost effective than IGRAs for LTBI screening. Our key views about the cost effectiveness of LTBI screening approaches in various subpopulations are highlighted in Box 1.

Box 1 **Key messages from economic evidence supporting LTBI screening.**

- Different economic evidence from different parts of the world has led to different national policies on LTBI testing concerning the diagnostic algorithm, frequency of testing and key populations that should be targeted for LTBI screening;
- Higher upfront unit cost of IGRAs compared to TST (due to increased cost of consumables, laboratory infrastructure and specialized training) means they are less likely to be cost effective as an LTBI screening strategy in LMICs. Economic evidence shows IGRAs are consistently cost effective in HICs where unit test costs are offset by averted TB treatment costs, although many countries prefer a sequential testing approach with IGRAs to reduce total overall costs;
- The requirement for sophisticated laboratory and human resource associated with IGRAs, in the context of the significant health infrastructure and personnel challenges frequently faced by LMIC health systems, makes the logistical and performance simplicity of TST a much more attractive, feasible and frequently more cost-effective LTBI screening option in LMIC settings;
- Evidence suggests targeted, IGRA-based LTBI screening in high-risk groups such as migrants, contacts and healthcare workers is cost effective, but cost effectiveness may not hold if screening is performed at lower TB incidence thresholds.

## 3. TB Preventive Treatment (TPT)

**Figure 3:**
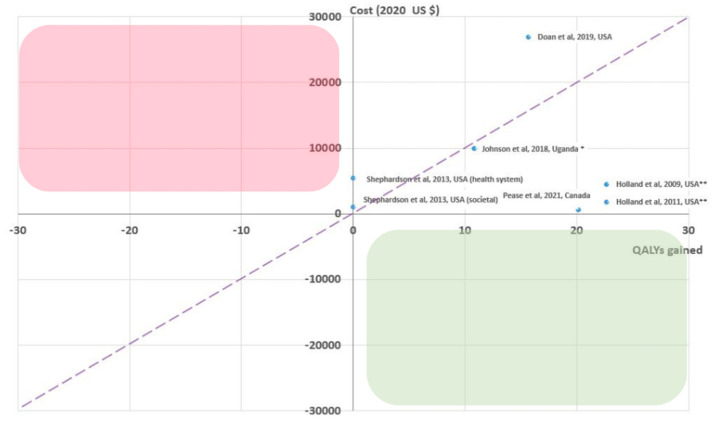
Incremental cost-effectiveness plane showing regimen specific ICERs comparing a novel shortened regimen for LTBI (3HP DOT) against a standard-of-care approach using 9H or 6H DOT or SA.* 2020 US $/DALY averted in LMIC ** ICERs calculated using 9H SAT as reference [34,35,36,37,38,39,40].

Scaling up TPT is a key pillar in the End TB Strategy but continues to be under-utilized due to limited resources and logistical challenges, with only a few high-TB-burden countries being successful in scaling up TPT activities per WHO recommendations [6,11,32]. Early work has shown that additional human and financial resources are required to scale up TPT implementation; therefore, economic evidence is critical in guiding scale-up [33]. Novel shortened treatment regimens introduced over the last decade hold great promise, with improved acceptability and feasibility and, in many cases, are leading to improved cost effectiveness or even cost savings compared to existing TPT regimens and programs. Figure 3 displays key cost-effectiveness estimates of TPT extracted from a recently published systematic review conducted by our group on the cost effectiveness of TPT regimens, all showing improved cost effectiveness of shortened TPT.

The most commonly used TPT regimens historically have been 6 or 9 months of self-administered isoniazid (6H or 9H); however, these regimens’ effectiveness is reduced by poor adherence rates and significant side effects. Consequently, shorter regimens such as 3 months of rifapentine and INH (3HP) taken once weekly and others, such as 4 months of rifampicin (4RIF), 3 months of INH and rifampicin (3HR) and 1 month of INH and rifapentine (1HP), have been more recently introduced and are being scaled up in many regions [35,41,42]. The new drug regimens themselves convey a higher cost compared to the inexpensive isoniazid [35], but the shorter duration of treatment coupled with potential for improved adherence has meant that these new approaches are cost effective and even provide cost savings in certain settings [35,36,37].

In one of the first cost-effectiveness analyses of shortened TPT, Shepardson et al. used data from a large U.S.-based trial and concluded that 3HP is a cost-effective alternative to self-administered 9H (ICER: USD 4565/QALY gained), particularly if the 3HP can be delivered by self-administered therapy (SAT) [36]. Doan et al. similarly found that directly observed treatment (DOT) with 3HP is a cost-effective TPT regimen with an ICER of USD 27,948/QALY gained compared to 3HP SAT. Doan also assessed 3HP SAT, 4R, 6H and 9H regimens compared to no treatment at all, finding all regimens to have fewer QALY gains than the 3HP SAT approach except for 3HP DOT [35]. Doan et al. also concluded that, for 3HP DOT to be cost effective relative to 3HP SAT, completion rates of at least 83% are required, which may be challenging to maintain in many settings with limited resources and shortages of healthcare personnel. In other settings, such as the Canadian arctic, where TB incidence rates are comparable to many parts of Sub-Saharan Africa, Pease et al. assessed the cost effectiveness of 3HP compared to 9H, finding 3HP to be dominant over 9H in this context where 9H is also routinely delivered through DOT. DOT using 3HP, therefore, is less costly and yields slightly more health gains compared with DOT using 9H [37]. 

Other regimens have also been found to be cost effective in certain settings. Pina et al. compared 4R to 6H amongst TB contacts in Spain, finding that 4R is a more cost-effective TPT regimen compared to 6H (ICER EUR 8737.86 per averted TB case) [43]. 1HP has emerged more recently as a potential TPT regimen, and, as such, economic evidence to support its use and rollout is more limited. Comparatively fewer economic studies on shortened TPT regimens have been conducted in LMICs, and, while that trend is recently changing, most studies of this type focused on assessing the cost effectiveness of TPT approaches in key high-risk groups such as PLHIV, household contacts and children, healthcare workers, migrants and those living in correctional facilities or other congregate living settings [8].

Despite being a priority group for LTBI screening and TPT, only one-third of eligible children were initiated on TPT in 2018 globally [44]. A 2020 study conducted by Jo et al. [44] across twelve high-TB-burden countries compared the cost effectiveness of three scenarios: (i) existing INH TPT coverage, (ii) contact case management with treatment of active TB but no TPT and (iii) contact case management coupled with treatment of active TB treatment and short-course TPT in children and adolescents under 15 years. They found that, across the board, contact case management and TPT using 3HP without LTBI testing is highly cost effective compared to the status quo of existing TPT coverage with INH (ranging from USD 100/DALY averted in Malawi to USD 1600 in Brazil) and superior to contact investigation and TB treatment without TPT [44].

Further research is needed to look into expanded contact case management interventions in children and HHC concerning feasibility, availability of resources for community-based screening visits, local HIV/TB co-infection prevalence and TB case fatality rates to increase the evidence base for policy decision making [44]. Box 2 outlines the authors’ main conclusions about the available economic evidence regarding TPT regimens in different populations and settings.

Box 2 **Key TPT message.**

- Despite higher drug regimen costs associated with newer shortened TPT regimens compared to the traditional TPT regimens, the potential for improved adherence and fewer side effects, leading to higher cure rates, makes the newer regimens a more attractive, economic option;
- Economic studies around shorter TPT regimens conducted in LMICs mostly targeted the use of these regimens in high-risk populations;
- Shortened TPT has been shown to be consistently highly cost effective in high-risk groups, namely, PLHIV, household contacts and children; this is true across both high- and low-TB-burden settings;
- Provision of shorter TPT regimens such as 3HP without LTBI screening is also highly cost effective among select high-risk groups such as PLHIV living in TB-endemic settings;
- Economic evidence is lacking to support and guide implementation and scale-up of TPT more widely in LMICs.

## 4. LTBI Screening and TPT among PLHIV

HIV increases the risk of progression of LTBI to active disease by between 20 and 26 fold [45], making this one of the most important groups for targeted LTBI screening and TPT. Targeted LTBI screening in PLHIV coupled with cost-effective TPT regimens can reduce the rate of progression of LTBI to active TB and, consequently, reduce community transmission of TB [7].

Transmission-modeling-based cost effectiveness analyses conducted at the state level in the U.S. showed that targeted LTBI screening and treatment approaches are most cost effective among PLHIV (ranging from US $2828 per QALY gained in Florida to US $11,265 per QALY gained in New York) and least cost effective among people with diabetes (ranging from US $223,041 per QALY gained in California to US $818,000 per QALY gained in New York) [46]. This is substantiated by other such analyses from HICs; Capocci et al. evaluated 30 testing approaches for LTBI and TB disease (symptomatic and asymptomatic) in a UK outpatient HIV clinic and found that testing is cost effective when targeting individuals from high-TB-burden countries in Sub-Saharan Africa or those from areas with a TB incidence greater than 40 per 100,000 at a willingness-to-pay threshold of GBP 30,000 per QALY gained [45].

When considering TPT among PLHIV, the literature is considerably broader, particularly within the LMIC space. In 2018, the WHO recommended 3 months of 3HP taken once weekly as a TPT option for PLHIV in high-TB-burden countries [47]. A recent systematic review and meta-analysis by Uppal et al. [11], concentrating on PLHIV, found over 40 studies from LMICs and concluded that TPT is universally cost effective among PLHIV compared to no TPT, regardless of the study setting or TPT regimen used. The cost effectiveness of TPT in PLHIV remained generally consistent across studies, despite the wide variability in the methodological approaches taken. 

Novel regimens such as 1HP taken weekly have been demonstrated to have similar cost effectiveness among PLHIV in the Ugandan setting with an ICER of US $1221 per DALY averted compared to 3HP. The cost effectiveness of this TPT regimen is estimated to be strongly linked to the price of rifapentine, local LTBI prevalence and completion rates. As the cost of rifapentine falls, evidence suggests we could see further improved affordability and cost effectiveness of rifapentine-based regimens [42]. 

## 5. Highlighting Key Gaps in Knowledge around LTBI Screening and Treatment Costs and Cost Effectiveness

Economic studies employ a range of different epidemiological parameters and key assumptions which may not hold across different populations. LTBI screening requires assumptions about imperfect gold standards, which poses challenges for any diagnostic accuracy study within LTBI. Cost-effectiveness analyses in LTBI require key assumptions around the number of averted TB cases driven by the risk of progression to active disease. The time horizon selected for analysis can also have an important impact on the cost effectiveness of LTBI interventions as cost effectiveness is often improved over longer time frames (allowing for more averted TB cases and, thus, more recouped cost savings). This heterogeneity in the methodological approach means comparisons and generalizations are challenging; however, when multiple economic evaluations conclude with consistent findings despite these methodological and setting specific differences, this helps us to feel comfortable making generalizations across studies and settings, such as in the case of PLHIV and TPT. This is not the case for LTBI screening more widely, where heterogeneity in modeling and cost-effectiveness results suggests that different approaches may be more suitable across different settings to maintain consistent cost-effectiveness standards.

As the scale-up of LTBI screening and TPT programs is expanded beyond more traditional high-risk groups, we should continue to invest in LTBI screening and TPT economic studies to be conducted across LMIC settings to better inform and guide these decisions by providing locally relevant evidence to drive sound economic policy.

Despite a large amount of heterogeneity in methodology across different cost-effectiveness analyses and settings, several findings were consistent. The cost effectiveness of LTBI screening programs is linked closely with the prevalence of LTBI, particularly LTBI due to recent transmission, and cost effectiveness is improved among populations with a higher risk of LTBI and/or higher risk of progression to active TB diseases such as PLHIV and household contacts. 

Novel shortened regimens have been consistently shown to be cost effective across a range of settings compared with 9H; however, key aspects of implementation such as ensuring high rates of adherence and completion are critical, and costs associated with adherence programs are not routinely included. Omission of these costs, particularly in LMIC contexts with already overburdened infrastructure and limited human resources for health, is likely to have dire consequences for the sustainability and impact of LTBI screening and TPT programs in these countries. Digital and other adherence support approaches are now being assessed for their utility and cost effectiveness in conjunction with novel shortened TPT regimens.

## 6. Conclusions

Economic evidence around scaling up interventions to target LTBI, including screening and TPT programs, is growing. While, historically, these analyses have focused on high-income, low-TB-incidence settings, more work is now being conducted in high-TB-incidence settings, reflecting an important shift in priorities and the recognition that investment in LTBI screening and treatment in LMICs is critical for reaching the End TB Strategy goals. Economic evidence is but one piece of the puzzle to inform decision making, and other factors should also be considered such as value preferences, access to care and other key equity issues.

## Data Availability

No new data were created or analyzed in this study. Data sharing is not applicable to this article.
